# Illicit Heroin and Methamphetamine Use among Methadone Maintenance Treatment Patients in Dehong Prefecture of Yunnan Province, China

**DOI:** 10.1371/journal.pone.0133431

**Published:** 2015-07-21

**Authors:** Rongrong Wang, Yingying Ding, Hongling Bai, Song Duan, Runhua Ye, Yuecheng Yang, Jibao Wang, Renhai Tang, Meiyang Gao, Na He

**Affiliations:** 1 Department of Epidemiology, School of Public Health, Fudan University, Shanghai, China; 2 The Key Laboratory of Public Health Safety of Ministry of Education, Fudan University, Shanghai, China; 3 Collaborative Innovation Center of Social Risks Governance in Health, Fudan University, Shanghai, China; 4 Department of Disease Control and Prevention, National Health and Family Planning Commission, Beijing, China; 5 Dehong Prefecture Center for Disease Control and Prevention, Mangshi, Yunnan Province, China; Temple University School of Medicine, UNITED STATES

## Abstract

**Objective:**

Methadone maintenance treatment (MMT) was introduced to China in 2004 to reduce the harm of injecting drug users (IDUs). However, little is known about continued drug use, especially methamphetamine (MAMP), among MMT patients.

**Methods:**

A survey was conducted among patients attending five major MMT clinics in Dehong Prefecture in 2014 to investigate the heroin and MAMP use and their associated risk factors. Participants were administered with face-to-face interviews, and urine tests for morphine and MAMP.

**Results:**

A total of 2,121 were eligible and participated in the study. Among them, 220 (10.4%) were only positive for morphine, 12.9% were only positive for MAMP, and 196 (9.2%) were positive for both morphine and MAMP. Compared with neither use of heroin nor MAMP during MMT, heroin use (not using MAMP) was associated with ethnicity, shorter duration of MMT, lower dose of methadone, and having had no more than two sex partners in the past year; MAMP use (not using heroin) was associated with ethnicity, longer duration of MMT, higher dose of methadone and being aged <30 years (vs. ≥50 years); use of both heroin and MAMP was associated with being Dai minority (vs. Han), a marital status of divorced or widowed, having used drugs for ≥10 years and shorter duration of MMT.

**Conclusion:**

These findings indicate the complexity in the treatment of heroin users and underscore the importance in prescribing appropriate methadone dosages in order to reduce both heroin and MAMP use.

## Introduction

Injection drug use (IDU), particularly heroin injection, contributes substantially to the spread of the HIV epidemic in China [1]. By the end of 2011, approximately 780,000 people were estimated to be living with HIV/AIDS nationwide, with 28.4% of infections attributed to IDU [2]. In order to address the growing IDU-driven HIV epidemic, the methadone maintenance treatment (MMT) was initiated as a pilot program in 8 clinics in 2004 in China and scaled up to 756 clinics nationwide cumulatively treating more than 384,500 drug users with 208 450 clients still receiving treatment by the end of 2012 [3,4]. There has been a growing body of literature indicating that MMT is effective in reducing heroin use, drug-related criminal activities, and HIV transmission as well as improving social well-being [5–8]. Nevertheless, many MMT patients continue using heroin while in treatment [9–11].

China recently experienced a significant increase in the use of new-type drugs, which refer to a type of drugs receiving popularity in a relatively short time as compared to opium and heroin. These drugs are often called as club drugs in western societies [12]. Methamphetamine (MAMP) was the most commonly abused new-type drug. By the end of 2012, there were approximately 0.41 million registered MAMP users, accounting for 23% of the 1.8 million registered drug users, second only to the heroin users [13]. The growing availability of MAMP raised serious concerns about its abuse among MMT patients [14], which has the potential to reduce the effectiveness of MMT program [15]. Furthermore, prior literature indicated that the use of MAMP is associated with increased risky sexual behaviors [16–18]. Given the high prevalence of HIV among IDUs [19], MAMP use will increase their risk of infecting or transmitting HIV through sexual activity.

The existing literature on illicit drug use during MMT program mainly focuses on studies of continued heroin use. Few studies focused on the methamphetamine use among MMT patients but all of them had small sample size, limiting the ability to identify the significant predictors [8, 9, 20]. To fill this gap, this study aimed to examine the heroin and MAMP use and their associated factors among MMT patients in Dehong Dai and Jingpo Autonomous Prefecture in Yunnan Province. The study area was chosen for the three primary reasons: first, as Dehong prefecture borders the drug-trafficking routes known as the “Golden Triangle”, it has a very large drug use population, and by the end of 2013, there were 12,000 officially registered drug users, accounting for approximately 1% of the total population in Dehong prefecture; second, Dehong recently experienced a rapid increase in MAMP use, which was significantly higher than the national level; third, the first HIV outbreak in China was reported among IDUs in 1989 in Dehong prefecture [21], where 44.6% of 14,270 reported HIV/AIDS cases were infected through IDU by the end of 2013.

## Methods

### Ethics Statement

This study was approved by the Institutional Review Board (IRB) of Fudan University, Shanghai, China.

### Study site and study participants

This cross-sectional survey was conducted in Dehong Dai and Jingpo Autonomous Prefecture in Yunnan Province. Participants were recruited from all of the five MMT clinics in Dehong prefecture in 2014. The eligibility criteria to participate in MMT included: 1) met the Chinese Classification of Mental Disorders version 3 criteria for opioid dependence, 2) aged at least 20 years, 3) registered as a local resident in the local area where the clinic was located, 4) had no contra-indications for taking methadone, and 5) agreed to the clinic rules [8, 11]. To be eligible for this study, the potential MMT patients had to provide informed consent to participate in the survey.

### Data collection

At enrollment, each participant completed a written informed consent and was interviewed face-to-face by a well-trained public health professional in a private place using a structured questionnaire. The questionnaire included items on demographic characteristics (including age, gender, education, and marital status), sexual behaviors (including number of sexual partners in the past year, sexual contacts in the past month and condom use), and history of drug use before MMT. Information about daily methadone dosage and the length of treatment was obtained from MMT Data System.

### Urine tests for heroin and methamphetamine

Urine samples were collected and tested immediately for morphine and MAMP by using colloidal gold rapid detection method (ABON Biopharm Co., Ltd, Hangzhou, China). A positive urine morphine result was considered as using heroin during MMT, as it was the most commonly abused opiate drugs in the study area and other opiate drugs were less common. A positive MAMP result was considered as using MAMP during MMT.

### HIV testing

Venous blood was collected for HIV testing. HIV infection was screened using using enzyme linked immunosorbant assay (ELISA) (Kehua Biotech, Shanghai, China). Any samples that screened positive for HIV was confirmed by Western blot (Genelabs Diagnostics, Singapore).

### Data analysis

All statistical analyses were performed using SAS software (Version 9.11, SAS Institute, USA). Categorical variables were compared using a chi-square test. Univariate and multivariate logistic regression models were used to examine factors associated with either heroin use or MAMP use or both during treatment, respectively. In multivariate analysis, we included variables with P values less than 0.10 in univariate analyses. Missing values were excluded from the corresponding analysis. A significance level of 0.05 was used for all tests.

## Results

### Sociodemographic characteristics

At the study period, a total of 2,600 adult drug users were receiving MMT in Dehong prefecture. Of them, 2,121 (81.6%) were eligible and participated in the study. Among them, 96.2% were males, 69% aged between 30–49, 70.5% were currently married, 58.3% were ethnic minorities, 50.1% were illiterate or educated only at primary school, and 77.0% were employed (Table 1).

**Table 1:** Characteristics of study participants according to urine test results for morphine and MAMP (*N* = 2121).

Characteristics	Urine Test											
	Total		Morphine(-)/MAMP (-)		Morphine(+)/MAMP (-)		Morphine(-)/MAMP (+)		Morphine(+)/MAMP (+)		*χ2*	*P*
	N	%	N	%	N	%	N	%	N	%		
**Demographics**												
Gender											0.8	0.834
Male	2041	96.2	1375	67.4	214	10.5	263	12.9	189	9.2		
Female	80	3.8	57	71.2	6	7.5	10	12.5	7	8.8		
Age (years)											32.9	**<0.001**
18–29	251	11.8	156	62.2	33	13.1	33	13.1	29	11.6		
30–39	811	38.2	536	66.1	73	9.0	128	15.8	74	9.1		
40–49	653	30.8	464	71.1	52	8.0	78	11.9	59	9.0		
50–79	406	19.2	276	68.0	62	15.3	34	8.4	34	8.4		
Ethnicity											58.5	**<0.01**
Han	885	41.7	620	70.0	90	10.2	109	12.3	66	7.5		
Dai	909	42.9	589	64.8	71	7.8	144	15.8	105	11.6		
Jingpo	249	11.7	168	67.5	50	20.1	13	5.2	18	7.2		
Others	78	3.7	55	70.5	9	11.5	7	9.0	7	9.0		
Marital status											16.0	**0.014**
Never married	378	17.8	251	66.4	39	10.3	56	14.8	32	8.5		
Currently married	1495	70.5	1026	68.6	161	10.8	182	12.2	126	8.4		
Divorced or widowed	248	11.7	155	62.5	20	8.1	35	14.1	38	15.3		
Education											10.3	0.111
Illiteracy or primary school	1063	50.1	703	66.1	109	10.3	133	12.5	118	11.1		
Middle school	816	38.5	555	68.0	89	10.9	109	13.4	63	7.7		
High school or above	242	11.4	174	71.9	22	9.1	31	12.8	15	6.2		
Occupation											14.4	**0.002**
Unemployed	488	23.0	353	72.4	30	6.1	66	13.5	39	8.0		
Employed	1633	77.0	1079	66.1	190	11.6	207	12.7	157	9.6		
**History of drug use**												
Age at first drug use (years)											15.1	0.002
<25	1200	56.6	810	67.5	109	9.1	179	14.9	102	8.5		
≥25	921	43.4	622	67.5	111	12.1	94	10.2	94	10.2		
Length of drug use (years)											17.9	**<0.001**
<10	542	25.5	337	62.2	75	13.8	65	12.0	65	12.0		
≥10	1579	74.5	1095	65.9	145	9.2	208	13.2	131	8.3		
Ever injected drugs											8.2	**0.042**
No	1365	64.4	901	66.0	159	11.6	173	12.7	132	9.7		
Yes	756	35.6	531	70.2	61	8.1	100	13.2	64	8.5		
**MMT experience**												
Length of MMT (years)											94.0	**<0.001**
<1	363	17.1	211	58.1	77	21.2	27	7.4	48	13.2		
1–5	1080	50.9	751	69.8	104	9.6	124	11.5	98	9.1		
>5	678	32.0	467	68.9	39	5.8	122	18.0	50	7.4		
Daily methadone dose (ml)											43.8	**<0.001**
≤30	335	16.4	224	66.9	56	16.7	26	7.8	29	8.7		
31–60	765	37.5	523	68.4	83	10.8	96	12.5	63	8.2		
61–99	528	25.9	362	26.1	40	7.6	77	14.6	49	9.3		
≥100	411	20.2	280	20.2	20	4.9	67	25.2	44	10.7		
**Sexual behaviors**												
No. of sexual partners in the past year											8.0	0.238
0	601	28.4	404	67.2	64	10.6	71	11.8	62	10.3		
1	1441	68.1	966	67.0	152	10.5	196	13.6	127	8.8		
≥2	75	3.5	59	78.7	3	4.0	6	8.0	7	9.3		
Condom use for sex in the past month											11.0	0.089
No sex	862	41.0	583	67.6	94	10.9	100	11.6	85	9.9		
Consistent condom use	313	14.9	222	70.9	19	6.1	47	15.0	25	8.0		
Inconsistent condom use	929	44.1	612	65.9	106	11.4	125	13.5	86	9.3		
**HIV serostatus**											13.2	**0.010**
Negative	1688	79.6	1128	66.8	194	11.5	213	12.6	153	9.1		
Positive	433	20.4	304	70.2	26	6.0	60	13.9	43	9.9		

### Sexual behaviors

About 71.6% (1516/2117) of the participants including 1456 males (71.5%) and 60 females (75.0%) reported having had sex in the past year. Among them, 75 (4.9%) participants including 70 (4.8%) males and 5 (8.3%) females had two or more sex partners in the past year. Approximately sixty percent (59.0%, or 1242/2104) of the participants had sexual behaviors in the past months with an average of 5 sexual intercourses, of whom 929 (74.7%) reported inconsistent condom use.

### HIV infection

Overall, 433 participants (20.4%) including 414 males and 19 females were tested HIV-positive. The HIV prevalence was not significantly different by gender (20.3% for males and 23.8% for females) and education level but was significantly different by age, ethnicity, marital status, occupation and history of drug use (data not shown).

### Methadone maintenance treatment experience

The participants had received MMT for an average of 3.8 years, ranging from 10 days to 11.8 years, with the majority (82.9%) having received MMT for at least one year (Table 1). The daily dose of methadone was 68.8 ml on average, less than 30 ml for 16.4% participants and more than 60 ml for 46.1% participants (Table 1).

### Illicit drug use before treatment

Participants used drugs for the first time at a mean age of 25.5±8.4 years, ranging from 6 to 63 years old, with 1200 (56.5%) participants including 1156 (56.6%) males and 44 (55.0%) females starting to use drugs prior to age 25. The mean length of illicit drug use before MMT was 15.6±8.0 years and most (74.5%) of the participants had used drugs for 10 years or more before MMT (Table 1). Almost all (98.0%) of the participants had only used heroin before MMT. More than one-third (35.6%, or 756/2121) of the participants including 35.4% (722/2041) of male participants and 42.5% (34/80) of female participants reported having injected drugs before MMT. Among them, 38.5% (278/722) males and 20.6% females (7/34) had shared needles.

### Heroin and methamphetamine use during treatment

A total of 2121 participants received urine tests for use of morphine and MAMP. Among them, 220 (10.4%) were only positive for morphine, 273 (12.9%) were only positive for MAMP, 196 (9.2%) were positive for both morphine and MAMP, and the remaining 1432 (67.5%) were negative for both morphine and MAMP. As shown in Table 1, illicit drug use during treatment was significantly associated with race, ethnicity, marital status, occupation, history of drug use before MMT, MMT experience and HIV infection status, according to chi-square tests.

Table 2 shows results of the multivariate logistic regression analysis for factors associated with use of either heroin or MAMP alone or both during treatment among the participants by controlling for potential confounding variables. Compared with neither use of heroin or MAMP during MMT program, participants of Jingpo minority (OR = 1.90, 95%CI: 1.23–2.95) were more likely to use heroin only during treatment (i.e., positive urine test for morphine only), whereas those of Dai minority (OR = 0.66, 95%CI: 0.45–0.96), having received MMT for a longer period (OR = 0.50, 95%CI: 0.34–0.72 for 1–5 years and OR = 0.37, 95%CI: 0.23–0.61 for >5 years), having received higher dose of methadone (OR = 0.64, 95%CI: 0.43–0.94 for 31–60 ml, OR = 0.47, 95%CI: 0.30–0.75 for 61–99 ml and OR = 0.36, 95%CI:0.20–0.64 for ≥100 ml), and having had two or more sexual partners in the past year (OR = 0.27, 95%CI: 0.08–0.92) were less likely to use heroin only during treatment.

**Table 2:** Multivariate logistic regression analysis for correlates of heroin and MAMP use among MMT patients.

Characteristics	Urine Test					
	Morphine(+)/MAMP(-)		Morphine (-)/MAMP(+)		Morphine(+)/MAMP(+)	
	Unadjusted OR (95%CI)	Adjusted OR (95%CI)	Unadjusted OR (95%CI)	Adjusted OR (95%CI)	Unadjusted OR (95%CI)	Adjusted OR (95%CI)
Gender (male vs. female)	1.48(0.63–3.47)		1.09(0.55–2.16)		1.12(0.50–2.49)	
Age (years)						
18–29	1.00	1.00	1.00	1.00	1.00	
30–39	0.64(0.41–1.01)	0.94(0.54–1.66)	1.13(0.74–1.72)	1.00(0.63–1.56)	0.74(0.47–1.18)	
40–49	**0.53(0.33–0.85)** **	0.82(0.43–1.58)	0.80(0.51–1.24)	0.66(0.40–1.08)	0.68(0.42–1.11)	
50–79	1.06(0.67–1.69)	1.41(0.70–2.84)	**0.58(0.35–0.98)** *	**0.50(0.28–0.92)** *	0.66(0.39–1.13)	
Ethnicity						
Han	1.00	1.00	1.00	1.00	1.00	1.00
Dai	0.83 (0.60–1.16)	**0.66 (0.45–0.96)** *	**1.39(1.06–1.83)** *	**1.63(1.22–2.16)** ***	**1.67(1.21–2.32)** **	**1.50(1.04–2.14)** *
Jingpo	**2.05(1.39–3.01)** ***	**1.90(1.23–2.95)** **	**0.44(0.24–0.80)** **	**0.47(0.25–0.86)** *	1.01(0.58–1.74)	0.84(0.48–1.48)
Others	1.13(0.54–2.36)	1.03(0.48–2.24)	0.72(0.32–1.63)	0.80(0.35–1.83)	1.20(0.52–2.73)	0.99(0.42–2.31)
Marital status						
Never married	1.00		1.00		1.00	1.00
Currently married	1.01(0.69–1.47)		0.80(0.57–1.11)		0.96(0.64–1.45)	0.88(0.57–1.34)
Divorced or widowed	0.83(0.47–1.48)		1.01(0.63–1.61)		**1.92(1.15–3.21)** *	**1.91(1.13–3.22)** *
Education level						
Illiteracy or primary school	1.00		1.00		1.00	1.00
Middle school	1.03(0.76–1.40)		1.04(0.79–1.37)		**0.68(0.49–0.94)** *	0.77(0.54–1.10)
High school or above	0.82(0.50–1.33)		0.94(0.62–1.44)		**0.51(0.29–0.90)** *	0.59(0.33–1.08)
Occupation (employed vs. unemployed)	**2.07(1.38–3.10)** ***	1.31(0.82–2.10)	1.03(0.76–1.39)		1.32(0.91–1.91)	
Age at first drug use (≥25 vs. <25 years)	1.33(0.99–1.76)	0.99(0.66–1.49)	**0.68(0.52–0.90)** *	0.99(0.71–1.37)	1.20(0.89–1.62)	
Length of drug use (≥10 vs. <10 years)	**0.60(0.44–0.81)** ***	0.87(0.56–1.34)	0.99(0.73–1.34)		**0.62(0.45–0.86)** *	**0.65(0.47–0.92)** *
Ever injected drugs (yes vs. no)	**0.65(0.48–0.89)** **	1.39(0.92–2.11)	0.98(0.75–1.28)		0.82(0.60–1.13)	
Length of MMT (years)						
<1	1.00	1.00	1.00	1.00	1.00	1.00
1–5	**0.38(0.27–0.53)** ***	**0.50(0.34–0.72)** ***	1.29(0.82–2.00)	1.31(0.81–2.11)	**0.57(0.39–0.83)** **	**0.60(0.40–0.88)** **
>5	**0.23(0.15–0.35)** ***	**0.37(0.23–0.61)** ***	**2.04(1.31–3.19)** **	**1.98(1.21–3.24)** **	**0.47(0.31–0.72)** **	**0.56(0.35–0.88)** *
Daily methadone dose (ml)						
≤30	1.00	1.00	1.00	1.00	1.00	
31–60	**0.64(0.44–0.92)** *	**0.64(0.43–0.94)** *	1.58(0.99–2.51)	**1.69(1.06–2.69)** *	0.93(0.58–1.48)	
61–99	**0.44(0.29–0.69)** ***	**0.47(0.30–0.75)** **	**1.83(1.14–2.94)** *	**1.86(1.15–3.02)** *	1.05(0.64–1.70)	
≥100	**0.29 (0.17–0.49)** ***	**0.36(0.20–0.64)** ***	**2.06(1.27–3.35)** **	**1.96(1.19–3.24)** **	1.21(0.74–2.00)	
No. of sexual partners in the past year						
0	1.00	1.00	1.00		1.00	
1	0.99(0.72–1.36)	0.95(0.67–1.35)	1.16(0.86–1.55)		0.86(0.62–1.19)	
≥2	0.32(0.10–1.05)	**0.27(0.08–0.91)** *	0.58(0.24–1.39)		0.77(0.34–1.77)	
HIV serostatus (positive vs. negative)	0.47(0.31–0.72)	**0.61(0.35–1.04)**	1.03(0.76–1.40)		1.04(0.73–1.49)	

*P<0.05

**P<0.01

***P<0.001.

Participants who were of Dai minority (OR = 1.63, 95%CI: 1.22–2.16), having received MMT for >5 years (OR = 1.98, 95%CI: 1.21–3.24) and received higher dose of methadone (OR = 1.69, 95%CI: 1.06–2.69 for 31–60 ml, OR = 1.86, 95%CI: 1.15–3.02 for 61–99 ml and OR = 1.96, 95%CI:1.19–3.24 for ≥100 ml methadone) were more likely to use MAMP only during treatment (i.e., positive urine test for MAMP only), whereas those who aged ≥50 years (OR = 0.50, 95% CI: 0.28–0.92) and were of Jingpo ethnicity (OR = 0.47, 95% CI: 0.25–0.86) were less likely to use MAMP only during treatment (Table 2).

Participants who were of Dai minority (OR = 1.50, 95%CI: 1.04–2.14), divorced or widowed (OR = 1.91, 95%CI: 1.13–3.22) were more likely to use both heroin and MAMP during treatment (i.e., positive urine test for both morphine and MAMP), whereas those who had used drugs for 10 years or more (OR = 0.65, 95% CI: 0.47–0.92), or had received MMT for longer period (OR = 0.60, 95% CI: 0.40–0.88 for 1–5 years; OR = 0.56, 95% CI: 0.35–0.88 for >5 years) were less likely to use both heroin and MAMP during treatment (Table 2).

## Discussion

Findings from the current analysis revealed that the prevalence of illicit drug use was high among MMT patients in Dehong prefecture, China. About one-fifth participants had a positive urine morphine test, which was slightly lower than that reported by a survey of 19,026 MMT patients in China [11]. It is likely that a number of MMT patients switch to use new-type drugs during treatment, as our data showed that about one-eighth participants were tested positive only for MAMP. Moreover, we found that about 9.2% patients were tested positive for both morphine and MAMP, which was consistent with previous studies in other areas of China reporting multiple drug use is common among MMT patients [9, 11].

An interesting finding was that the type of illicit drugs used during MMT program was related to ethnicity: compared to Han, Jingpo were more likely to use heroin but less likely to use MAMP, and Dai were more likely to use MAMP but less likely to use heroin. This may be due to the availability of the specific type of drugs, but this speculation requires further investigation.

We found that compared to those having received MMT for more than one year were less likely to use heroin than those having received MMT for less than one year. This was consistent with findings from previous studies that the proportion of positive urine result for morphine was high in the first year of MMT, after which it decreased [9, 22], and longer duration of MMT was associated with reduced heroin use [22, 23].

We found that lower methadone dose was associated with continued heroin use during treatment. The majority of previous findings, including clinical trials and observational studies, agreed that high methadone dosage (>60 mg) was more effective than lower doses (1 to 39 mg/day) in reducing illicit use of heroin [24–27]. On the contrary, only few studies found that low doses of methadone are associated with reduced heroin use. The dosage is often prescribed according to the patients’ addiction severity, resulting in lower dosage for less severe addiction[10,11].

A notable finding was that in contrast to heroin use, MAMP use during treatment was particularly common among patients who remained in MMT for a longer period and received higher dose of methadone. This is contrary to prior study indicating that high dose of methadone reduce the current use of another stimulant drug like cocaine during treatment [28]. But this finding supported Trujillo et al. who demonstrated that the combination of MAMP and methadone produce greater effects than either drug alone and reduce the side effects of each other [29]. A recent qualitative study explored the reasons for MMT patients co-using MAMP with methadone, including self-medication from methadone dependence, countering side-effects of methadone (e.g., impaired sexual performance, depression), increasing pleasure, and getting high [30]. This may explain the reasons why MMT participants co-use MAMP with methadone. The emergence of MAMP among MMT patients brings new challenges to current MMT programs and highlights the needs for psychological interventions and revisions for treatment protocols [30].

It is not surprising that those continued to use heroin during treatment were less likely to report having two or more sex partners in the past year. Heroin has been found to decrease sexual function [31]. We also found that HIV infection was associated with reduced heroin use during MMT program. This was a cross-sectional study; we cannot determine the causal relationships. It is possible that reduced heroin use during treatment was associated with decreased risk of HIV infection, as previous literature indicated [8].

This study has several limitations. First, this was a cross-sectional survey, we are not able to make any causal inference. Second, we have used self-reported data for the number of sex partners, age at first drug use, etc.; thus, recall or social desirability biases might be present. Third, the urine tests can only identify the heroin or MAMP use within the last 7 days, which might underestimate the actual prevalence of heroin and MAMP use among MMT patients. Finally, our participants were recruited from Dehong, the observed prevalence of illicit drug use in our study might not be generalizable to MMT patients in other areas. However, the factors associated with heroin and/or MAMP use during MMT and the dose of methadone, are consistent with previous studies [22–27, 29]; and thus these findings should not be severely affected. In summary, our study confirmed the positive effects of the long duration of MMT and high dose of methadone on reducing concurrent heroin use. But we found high prevalence of MAMP use among MMT patients, particularly among those remained in MMT for a longer period and received a high dose of methadone. These findings indicate the complexity in the treatment of heroin users and underscore the importance in prescribing appropriate methadone dosages in order to reduce both heroin and MAMP use. Further research is needed to explore the reasons for MAMP use by MMT patients in China and to develop effective intervention programs.
